# Comparative evaluation of extraction methods for apoplastic proteins from maize leaves

**DOI:** 10.1186/1746-4811-7-48

**Published:** 2011-12-22

**Authors:** Katja Witzel, Muhammad Shahzad, Andrea Matros, Hans-Peter Mock, Karl H Mühling

**Affiliations:** 1 Institute of Plant Nutrition and Soil Science, Christian Albrechts University, Hermann-Rodewald-Strasse 2, 24118 Kiel, Germany; 2 Leibniz Institute of Plant Genetics and Crop Plant Research, Corrensstrasse 3, 06466 Gatersleben, Germany; 3 Leibniz Institute of Vegetable and Ornamental Crops, Theodor-Echtermeyer-Weg 1, 14979 Großbeeren, Germany

**Keywords:** Apoplast, liquid chromatography mass spectrometry, maize, proteome analysis, two-dimensional gel electrophoresis

## Abstract

Proteins in the plant apoplast are essential for many physiological processes. We have analysed and compared six different infiltration solutions for proteins contained in the apoplast to recognize the most suitable method for leaves and to establish proteome maps for each extraction. The efficiency of protocols was evaluated by comparing the protein patterns resolved by 1-DE and 2-DE, and revealed distinct characteristics for each infiltration solution. Nano-LC-ESI-Q-TOF MS analysis of all fractions was applied to cover all proteins differentially extracted by infiltration solutions and led to the identification of 328 proteins in total in apoplast preparations. The predicted subcellular protein localisation distinguished the examined infiltration solutions in those with high or low amounts of intracellular protein contaminations, and with high or low quantities of secreted proteins. All tested infiltration solution extracted different subsets of proteins, and those implications on apoplast-specific studies are discussed.

## Background

The plant apoplast comprises the cell wall matrix and the intercellular spaces, and plays a major role in a wide range of physiological processes, including water and nutrient transport [1], plant-pathogen interactions, and perception and transduction of environmental signals [2,3]. Proteins present in the plant apoplast reflect this broad functional diversity. Studies on the dynamic change of apoplast protein composition revealed new insights into plant responses to abiotic stress [4-7], nutrient supply [8-10], wounding [11], water deficiency [12,13], pathogen response [14-16] and xylem composition [17,18]. The selection of a suitable extraction protocol is a crucial step in proteomics surveys as proteins reveal a high degree of biochemical heterogeneity and investigated plant materials can be characterized by the presence of non-protein components interfering with subsequent analytical techniques, e.g. two-dimensional gel electrophoresis (2-DE) or liquid chromatography-mass spectrometry (LC-MS). These biological realities led to the establishment of sample preparation methods for numerous plant species and tissues, such as Arabidopsis leaves [19], papaya leaves [20], sunflower leaves [21], cotton seedlings [22], apple and strawberry fruit [23], potato tuber [24], grapevine leaves and roots [25], grape berry cell wall [26], rubber latex [27], cotton fibers [28], banana meristem [29] and chloroplast [30], among others. Despite their biological significance, investigations on apoplastic proteins are hampered due to their low abundance compared to intracellular protein concentrations. The extraction of proteins from the leaf and root apoplast is mainly based on the principle of vacuum infiltration with an extraction solution, followed by a mild centrifugation step to collect the apoplastic washing fluid. The composition of the infiltration solution is essential as it has to fulfil certain prerequisites, such as maintenance of osmotic pressure to prevent collapsing of plasma membrane and stringency for extracting cell wall-bound proteins. Borderies et al. [31] compared different solutions to extract loosely bound cell wall proteins of Arabidopsis cell suspension cultures and showed that the composition of extraction solution determines the efficiency of preparation. Similarly, Boudart et al. [32] investigated weakly cell wall-bound proteins in rosettes of Arabidopsis. Here, we compared protein extracts obtained by six different infiltration solutions already described for apoplastic proteins from different plant species. We aimed at identifying a protocol most suitable for the extraction of leaf apoplast proteins of maize, a crop of high economic importance. We evaluated the protein patterns as resolved by 1-DE or 2-DE, identified the proteins using LC-MS and located them to cellular compartments.

## Results and discussion

**Figure 1:**
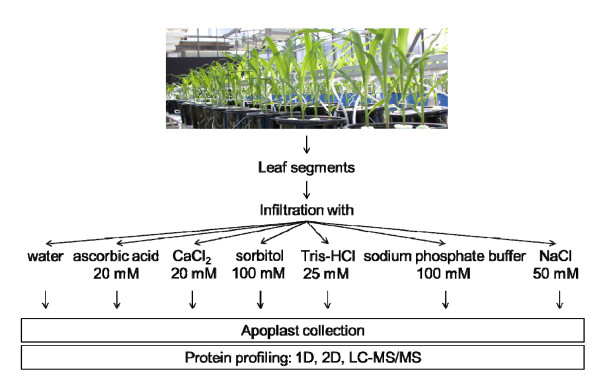
**Schematic representation of protein extraction from maize leaf apoplast**. Different infiltration solutions were analyzed for their specificity by proteome profiling using gel-based and gel-free approaches.

In this study, six different solutions were tested for the ability to extract proteins from the maize leaf apoplast: water [8], 20 mM ascorbic acid/20 mM CaCl_2 _[6], 100 mM sorbitol [4], 25 mM Tris-HCl [9], 100 mM sodium phosphate buffer [16] and 50 mM NaCl [33] (Figure 1). In most cases, the infiltration solutions were applied for wheat leaves and no comparison of the efficiency of protein extraction for each method was performed. Thus, this study focussed on identifying the optimal method for extracting apoplastic proteins from maize leaves.

**Figure 2:**
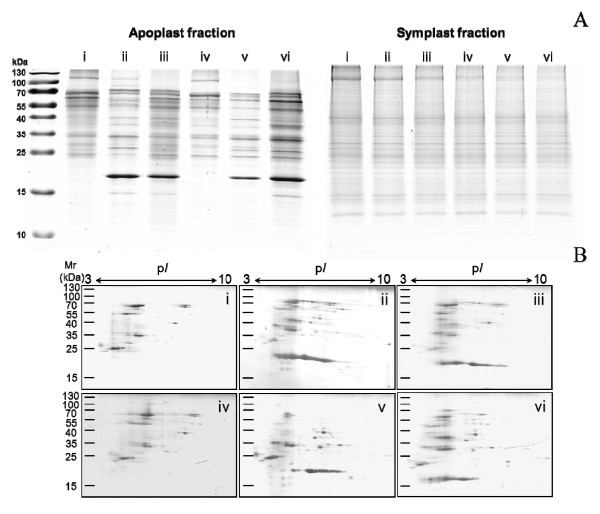
**Profiles of maize leaf protein extracts as resolved by 1-DE (A) and 2-DE (B)**. A: SDS-PAGE of apoplastic and symplastic proteins extracted with water (i), 100 mM sodium phosphate buffer (ii), 25 mM Tris-HCl (iii), 100 mM sorbitol (iv), 20 mM ascorbic acid/20 mM CaCl_2 _(v) or 50 mM NaCl (vi). A total of 10 μg protein per lane was loaded. B: 2-DE profiles of protein extracts from the maize leaf apoplast isoelectric focussed on IPG 3-10 and visualized by Coomassie staining. A total of 25 μg protein per gel was loaded.

Proteins from the leaf apoplast and symplast extracted with the six infiltration solutions were compared on 1-DE (Figure 2A, Additional file 1). A sharp band pattern was obtained from all apoplast extracts with a high number of protein bands in each extract. While the yield of protein extraction was similar, the protein profiles showed distinct differences. A prominent band of about 20 kDa was present in extracts of 100 mM sodium phosphate buffer, 25 mM Tris-HCl, 20 mM ascorbic acid/20 mM CaCl_2 _and 50 mM NaCl, but not in water or 100 mM sorbitol. One protein band of high molecular weight (approximately 100-130 kDa) was apparent in extracts of water, 100 mM sodium phosphate buffer and 100 mM sorbitol, but not in 25 mM Tris-HCl, 20 mM ascorbic acid/20 mM CaCl_2 _or 50 mM NaCl. While there were similarities, each extract revealed specific protein bands indicating that different subsets of proteins were isolated by the six infiltration solutions. Proteins with a molecular weight < 15 kDa were underrepresented in all extracts and this corresponds to previous proteomic reports on some of the infiltration solutions [4,16]. The observed selective protein patterns generated by the individual infiltration solutions emphasize the necessity of careful selection of isolation method [34]. Band patterns from symplast preparations did not reveal significant differences among the infiltrates and the overall band patterns were more complex as from apoplastic preparations. This demonstrates an apparent subfractionation of the cellular compartments.

Equal amounts of apoplast proteins were separated by 2-DE to assess the protein patterns in more detail (Figure 2B). We found areas of good and poor resolved proteins spots on all 2-D gels. Proteins in the acidic gel region of pH 4-6 showed horizontal streaking. Although all samples were precipitated, dissolved in urea-containing buffer system and dialyzed prior to 2-DE to avoid the contamination with nucleic acids or other interfering substances, these poorly separated spots were observed. Contrary to this, proteins in the basic region of 2-D gels near the pH 6-10 interval showed a superior resolution with minimal streaking. The spot patterns resembled the band patterns to a certain extent, e.g. as observed for the 20 kDa band that was prominent also on 2-D gels of the respective apoplastic extracts. The best resolution of proteins in 25-45 kDa intervals was achieved on extracts of 20 mM ascorbic acid/20 mM CaCl_2 _infiltration solution, while high molecular weight proteins separated best in extracts of 100 mM sodium phosphate buffer infiltration solution. The latter was applied with success to extract proteins from the leaf apoplast of lupin and resulted in the generation of well resolved protein maps containing about 50 spots to evaluate the effect of water and boron deficiency [9]. Our results showed that this separation was not reached, probably due to substances present in the maize apoplast interfering with isoelectric focusing. As 2-DE did not result in a comprehensible evaluation of the employed infiltration solutions, we used nano-LC-ESI-Q-TOF MS for proteomic analysis of all extracts.

In order to obtain an overview of all proteins present in the six different extracts, we aimed at establishing qualitative protein profiles by LC-MS analysis. An automatic data directed analysis mode was applied as described in materials and methods section. Results exceeding the PLGS score of 12 for protein identification and probability score of 50% for *de novo *sequencing of peptides were accepted.

**Figure 3:**
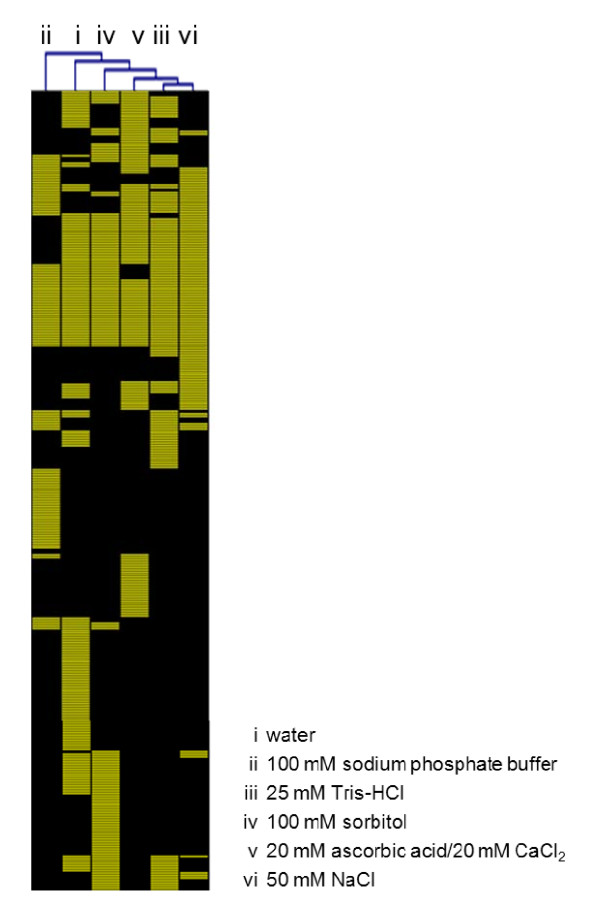
**Hierarchical clustering analysis of protein abundance patterns**. Columns represent LC-MS experiments on protein extracts of indicated infiltration solutions. Rows display the presence (yellow) or absence (black) of proteins in the respective extracts. Additional file 2 provides protein identifications and their detection in the respective apoplast extracts.

A total of 328 proteins were identified from all extracts. Additional file 2 shows the identities of those proteins, along with the predicted subcellular localization and detection in the six apoplastic extracts. Additional file 3 provides the respective identifier, PLGS score, number of peptides, protein coverage, peptide sequences and peptide sequence probability score for all identified proteins. In order to visualize and identify infiltration solutions with similar protein abundance patterns, a hierarchical clustering method was applied. Two main clusters were found, with the first represented by the 100 mM sodium phosphate buffer and the second containing all other infiltration solutions indicating the isolation of a rather different set of proteins by the first one than compared to all other solutions under examination (Figure 3). The most similar abundance patterns derived from leaf infiltration with 25 mM Tris-HCl and 50 mM NaCl reflecting a comparable degree of protein extraction efficiency.

The highest number of proteins was found in apoplastic extracts using water as infiltration solution. Here, 171 proteins were detected. Extracts of 25 mM Tris-HCl, 100 mM sorbitol and 20 mM ascorbic acid/20 mM CaCl_2 _yielded in the identification of a similar number of 131, 133 and 133 proteins, respectively. We found 114 proteins in extracts of 50 mM NaCl solution and 107 proteins in those of 100 mM sodium phosphate buffer. Out of all 328 proteins, only 28 proteins were common across all six extracts (Additional file 4). A similar observation was made for Arabidopsis cell wall proteins when extracted by different solutions; here, only 11 out of 96 proteins were found to be common in all extracts [31]. Exhydrolase II [UniProt: Q9XE93] was found in all extracts and its identification is illustrated in Additional file 5 as an example. Here, the amino acid sequence is shown and the 12 detected peptides are marked within, resulting in protein sequence coverage of 28.7%.

**Figure 4:**
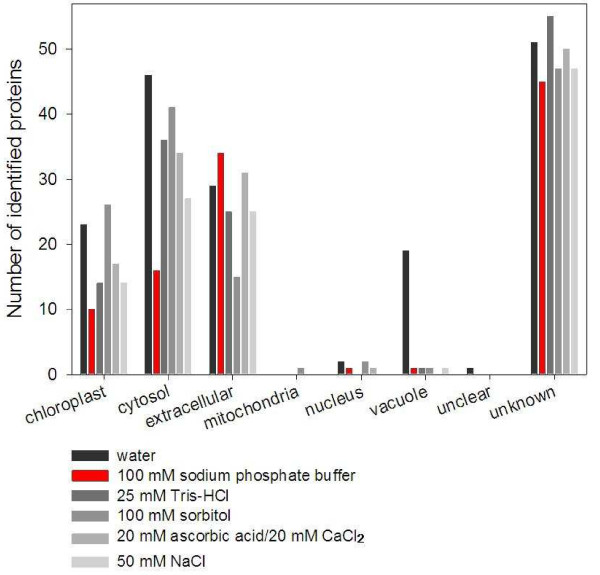
**Predicted subcellular distribution of identified proteins from the maize leaf apoplast extracted by different infiltration solutions**. Topology prediction was performed with Expasy tools Target P (http://www.cbs.dtu.dk/services/TargetP/) and WoLF PSORT (http://wolfpsort.org/).

The quality of apoplastic protein preparations is estimated in many cases by enzymatic measurements of specific proteins such as malate dehydrogenase [5,9] and glucose-6-phosphate dehydrogenase [6]. However, it is known that the activity of those enzymes is detectable in respective cellular compartment as well [35]. To assess the amount of symplast contaminations in our samples, we used topology prediction tools. The identified proteins were classified for their subcellular localization as deduced by Expasy tools Target P and WoLF PSORT (Figure 4). A number of proteins in this study were allocated to other cellular compartments then the apoplast, suggesting considerable amounts of intracellular protein contaminations. However, previous reports using different plant species and extraction methods described the detection of cytosolic, mitochondrial or vacuolar proteins in cell wall or apoplast preparations [31,36-38]. These consistent findings point to the occurrence of non-classical secretory pathways for proteins lacking signal sequences [39,40] and therefore, differentiation between yet unknown apoplastic proteins and ones resident in other organelles remains difficult. Water-infiltrated leaves revealed 23 protein identifications localized to the apoplast, while a high number of intracellular proteins were detected from the vacuole (19), cytosol (46) and chloroplast (23). This observation is indicative for the disrupture of plasma membrane during the infiltration process. Also, apoplastic extracts with 100 mM sorbitol as infiltration solution contained a superior proportion of chloroplast (26) and cytosolic (41) proteins with only 15 predicted apoplastic proteins. This result was unexpected as the sugar alcohol sorbitol was applied to maintain the osmotic cell pressure. Similar numbers of proteins in infiltrates with 25 mM Tris-HCl, 20 mM ascorbic acid/20 mM CaCl_2 _and 50 mM NaCl were assigned to the chloroplast (14, 17, 14), the cytosol (36, 34, 27), and the apoplast (25, 31, 25). Of all tested infiltration solutions, 100 mM sodium phosphate buffer contained the lowest number of proteins assigned to intracellular compartments (chloroplast: 10, cytosol: 16) and the highest number of proteins targeted to the extracellular apoplast with 34 identified proteins.

Table 1 presents the 67 proteins allocated to the apoplast of maize leaves and grouped according to their function into 7 classes. The largest class consisted of 39 proteins related to cell structural processes, including carbohydrate metabolism (e.g.: lichenase 2, alpha N-arabinofuranosidase, beta galactosidase, exoglucanase, exhydrolase II) and cell wall modification (e.g.; pectinesterase, xyloglucan endotransglycosylase hydrolase, peroxidases). Synthesis and integration of polysaccharides into the cell wall and extension of this network during plant growth are the major biological functions of proteins present in the apoplast [41] and our findings reflect this reality. Fifteen proteins were involved in disease and defense reactions, the second prime function of the apoplast [42]. The third class was related to proteins with transporting function and here, 7 proteins were identified. Further classes were related to cell growth/division, protein destination/storage, secondary metabolism and signal transduction.

**Table 1:** Identification of proteins assigned to the apoplast.

				Infiltration solutions					
				
Entry	Description	mW (Da)	pI (pH)	i	ii	iii	iv	v	vi
Cell growth and division									
UniRef90_Q6ZDE3	Abscisic acid 8'-hydroxylase 2, Oryza sativa	56519	9.83	x					
Cell structure									
UniRef90_A5H454	Peroxidase 66, Zea maize	33398	8.02					x	
UniRef90_A5H8G4	Peroxidase 1, Zea maize	38330	6.89					x	
UniRef90_A5JTQ2	Alpha N arabinofuranosidase, Medicago varia	83673	6.22		x				
UniRef90_B4FKV6	Peroxidase 54, Zea maize	36178	4.95						x
UniRef90_B5AK47	Dhurrinase-like B glucosidase, Zea maize	64233	7.95			x			
UniRef90_B6SMR2	Peroxidase 52, Zea mays	33504	8.14		x	x		x	x
UniRef90_B6SUH6	Non-cyanogenic beta glucosidase, Zea mays	56680	5.39	x		x		x	
UniRef90_B6SXU7	Heparanase-like protein 3, Zea maize	58262	9.35	x		x	x	x	x
UniRef90_B6SXY3	Beta galactosidase, Zea mays	48728	8.38		x				
UniRef90_B6T391	Lichenase 2, Zea mays	34951	5.64	x	x	x	x	x	x
UniRef90_B6T9B9	Alpha N arabinofuranosidase, Zea mays	74827	5.04			x		x	x
UniRef90_B6TU39	Peroxidase 2, Zea maize	34941	4.67		x				
UniRef90_B6TU78	Glucan endo-1-3 beta glucosidase 7, Zea maize	45316	5.54	x					
UniRef90_B6TXJ8	Glycoside hydrolase family 28, Zea maize	47066	5.58	x					
UniRef90_B6U063	Carboxylic ester hydrolase, Zea mays	50032	7.85	x		x		x	
UniRef90_B6U0W2	Beta galactosidase, Zea maize	93935	6.48		x				
UniRef90_B9SD68	Hydrolase, Ricinus communis	67825	6.80	x	x	x			
UniRef90_C4N559	Xyloglucan endotransglycosylase hydrolase, Musa acuminata	20309	9.54						x
UniRef90_C5WQU7	Beta galactosidase, Sorghum bicolor	92893	5.31	x		x	x	x	x
UniRef90_C5Z534	Beta galactosidase, Sorghum bicolor	79098	7.74		x				
UniRef90_O04943	Alpha galactosidase, Hordeum vulgare	17730	6.33		x	x		x	x
UniRef90_P93518	PRm 3, Zea maize	30099	3.90	x	x	x	x	x	x
UniRef90_Q10CU3	Glycosyl hydrolase family 3, Oryza sativa	43916	8.20	x		x			
UniRef90_Q10M79	Alpha L arabinofuranosidase, Oryza sativa	73965	4.73					x	
UniRef90_Q10NX8	Beta galactosidase 6, Oryza sativa	92780	5.52	x			x		
UniRef90_Q2R3E0	Glycosyl hydrolases family 38, Oryza sativa	114085	5.85		x				x
UniRef90_Q2RAZ2	Alpha L arabinofuranosidase, Oryza sativa	73421	4.57	x		x	x	x	x
UniRef90_Q43417	Peroxidase, Cenchrus ciliaris	32473	7.50					x	
UniRef90_Q53MP2	Beta D-xylosidase, Oryza sativa	82557	6.62		x				
UniRef90_Q5CCP6	Beta galactosidase, Pyrus pyrifolia	94782	8.12		x				
UniRef90_Q5I3F3	Peroxidase 5, Triticum monococcum	27533	5.72		x				
UniRef90_Q6L619	Beta galactosidase, Raphanus sativus	92580	8.36	x				x	
UniRef90_Q7G3T8	Beta galactosidase 13, Oryza sativa	91940	6.06		x				
UniRef90_Q8GUY1	Pectinesterase, Lolium perenne	24837	7.81		x				
UniRef90_Q8RUV9	Beta galactosidase 1, Oryza sativa	91652	5.71		x				
UniRef90_Q9FXT4	Alpha galactosidase, Oryza sativa	45792	7.91					x	x
UniRef90_Q9LLB8	Exoglucanase, Zea mays	66900	6.99	x	x	x	x		x
UniRef90_Q9XE93	Exhydrolase II, Zea mays	68330	6.16	x	x	x	x	x	x
UniRef90_Q9XEI3	Beta D-glucan exohydrolase isoenzyme, Hordeum vulgare	67862	6.24	x	x		x		
Disease and defence									
UniRef100_P25272	Kunitz-type trypsin inhibitor 1, Glycine max	22531	4.77	x					
UniRef90_A7IZL3	Invertase inhibitor, Coffea canephora	20205	6.68		x				
UniRef90_B6TA80	Thaumatin-like protein, Zea mays	17632	6.75					x	
UniRef90_B6TDW7	Secretory protein, Zea mays	24467	4.64					x	
UniRef90_B6TT00	Endochitinase PR4, Zea maize	28545	4.96	x				x	
UniRef90_B6TTY1	Germin-like protein, Zea maize	26764	7.25	x		x		x	x
UniRef90_B6TWH6	Lysosomal Pro × carboxypeptidase, Zea mays	59936	5.62	x	x	x	x		x
UniRef90_B6UB57	Lysosomal protective protein, Zea maize	53540	5.87	x	x	x		x	x
UniRef90_O24007	Chitinase, Oryza sativa	18956	4.83	x		x	x	x	x
UniRef90_P01063	Bowman-Birk-type proteinase inhibitor C II, Glycine max	9194	4.38	x					
UniRef90_P29022	Endochitinase A, Zea maize	29105	7.85					x	
UniRef90_Q5U1S9	Class III peroxidase 14, Oryza sativa	37174	5.77					x	
UniRef90_Q6EUS1	Class III peroxidase 27, Oryza sativa	33300	8.09		x				x
UniRef90_Q6TM44	Germin-like protein, Zea mays	21873	6.04	x	x	x	x	x	x
UniRef90_Q7M1R1	Chitinase, Gladiolus × gandavensis	30695	5.77	x		x	x	x	x
Protein destination and storage									
UniRef90_B6TG95	Vignain, Zea mays	38823	4.68	x	x	x	x	x	x
UniRef90_B6TYX7	Polygalacturonase inhibitor 1, Zea mays	30011	8.08		x			x	x
Secondary metabolism									
UniRef90_O64411	Polyamine oxidase, Zea mays	56308	5.63		x				
Signal transduction									
UniRef90_B6TWC3	Rhicadhesin receptor, Zea mays	23726	9.20		x	x			
UniRef90_B9MZ47	Fasciclin-like AGP 14 4 protein, Populus trichocarpa	24786	8.63					x	
Transporters									
UniRef90_B4FB54	Non-specific lipid transfer protein, Zea mays	12084	9.60		x				
UniRef90_B6SP11	Non-specific lipid transfer protein, Zea mays	9802	8.73		x				
UniRef90_B6SY96	Non-specific lipid transfer protein, Zea mays	12011	9.29		x				
UniRef90_B6TRB2	Copper ion binding protein, Zea maize	17057	9.78			x			
UniRef90_P05046	Lectin, Glycine max	30908	5.60	x			x		
UniRef90_P19656	Non-specific lipid transfer protein, Zea mays	11697	8.74		x				x
UniRef90_Q04672	Sucrose-binding protein, Glycine max	60484	6.42	x					

UniProt database identifiers, along with molecular weight (mW) and isoelectric point (pI) are shown. The identification of the respective proteins using different extraction solutions is indicated (i: water, ii: 100 mM sodium phosphate buffer, iii: 25 mM Tris-HCl, iv: 100 mM sorbitol, v: 20 mM ascorbic acid/20 mM CaCl_2_, vi: 50 mM NaCl).

The number of proteins identified exclusively in any of the extracts was compared and revealed that 16 out of 34 apoplastic proteins were found only in extracts of 100 mM sodium phosphate buffer, representing the highest number of unique proteins in all tested infiltration solutions (Table 1). Usage of this infiltration solution appears to prevent damaging the plasma membrane and enables extraction of proteins adhesive to the cell wall. Most polypeptides found in the analysis were annotated as hypothetical based on an *in silico *match to a genome sequence, or putative due to a homology to a protein with known function (Figure 4). The identification of these proteins in apoplastic preparations reveals the potential inherited in proteomic surveys for establishing comprehensive maps of all translated polypeptides present in a subcellular compartment. A number of 12 proteins with unknown function were exclusively identified using the 100 mM sodium phosphate buffer infiltration solution (see Additional file 2). As this protein fraction performed best regarding contaminations from other cellular compartments and contained most of the apoplastic proteins, we assume that these yet unknown proteins are involved in physiological processes of the apoplast.

## Conclusions

The plant apoplast is a dynamic compartment with a broad range of physiological functions. To study proteins involved in nutrition, growth, signaling or transport processes, it is crucial to apply extraction methods selective for apoplastic proteins. In this study, we compared six different infiltration solutions already reported for the isolation of this protein subset. The protein patterns resolved by 1-DE revealed clear differences between apoplast and symplast preparations. We found the lowest number of intracellular protein contaminants with the highest number of extracted proteins present in the apoplastic fluid obtained with 100 mM sodium phosphate buffer. Also, the number of secreted proteins exclusively found in a single fraction was highest for that buffer. Those findings are now employed in comparative proteomic studies aiming at identifying proteins involved in abiotic stress responses.

## Materials and methods

### Plant cultivation

Maize grains cv. Lector (LG Seeds, http://www.lgseeds.com) were imbibed overnight in aerated 1 mM CaSO_4 _solution and germinated at 28°C in the dark between two layers of filter paper moistened with 0.5 mM CaSO_4_. After 4 days, seedlings were transferred to light in constantly aerated plastic pots containing one-fourth concentrated nutrient solution. The concentration of nutrient solution was increased to half and full strength after 2 and 4 days of cultivation, respectively. The full strength nutrient solution had the following concentrations: 2.0 mM Ca(NO_3_)_2_, 1.0 mM K_2_SO_4_, 0.2 mM KH_2_PO_4_, 0.5 mM MgSO_4_, 2.0 mM CaCl_2_, 5.0 μM H_3_BO_3_, 2.0 μM MnSO_4_, 0.5 μM ZnSO_4_, 0.3 μM CuSO_4_, 0.01 μM (NH_4_)_6_Mo_7_O_24_, 200 μM Fe-EDTA. Nutrient solution was changed twice a week to avoid nutrient deficiencies. The experiments were carried out under greenhouse conditions with an average day/night temperature of 28/18°C and a photoperiod of 14 h for 5 weeks with relative humidity about 70% ± 5%. The fifth and sixth leaf from medium part of the stem was harvested 16 d after reaching the full nutrient solution for collection of apoplast proteins.

### Extraction of apoplastic and symplastic proteins

Apoplastic proteins were collected using the infiltration-centrifugation technique [43] with minor modifications. Leaves were cut into segments of about 5.5 cm and washed with deionised water. For infiltration, leaf segments were placed in plastic syringes (60 ml) filled with 40 ml of the respective infiltrating medium and were infiltrated by pulling the plunger, producing a reduced pressure of estimated about 20 kPa. Leaves were infiltrated either with water, 20 mM ascorbic acid/20 mM CaCl_2_, 100 mM sorbitol, 0.1 M sodium phosphate buffer (pH 6.5), 25 mM Tris-HCl (pH 8.0) or 50 mM NaCl (Figure 1). Thereafter, intact leave segments were carefully blotted dry, and then placed in a 10 ml plastic vessel and centrifuged immediately at 400 *g *for 5 min at 5°C. The clear infiltrate, now referred to as apoplast fraction, was collected at the bottom of the tube.

After the extraction of the apoplastic fraction, the residual leaf tissue was shock frozen in liquid nitrogen, thawed, and centrifuged at 715 *g *for 5 min for cell sap extraction, now referred to as symplast fraction. Four pools of extracts from five plants each were combined for subsequent analyses. Extracts were stored at -80°C until analysis.

### Gel electrophoretic protein separation

Proteins contained in the different extracts were precipitated by chloroform/methanol method [44]: 200 μl of sample was mixed with 800 μl MeOH, 400 μl chloroform and 600 μl deionized water. The incubation at 4°C for 5 min was followed by a centrifugation step (9,000 *g*, 2 min, 4°C). The upper phase was removed and 600 μl MeOH was added to the lower and interphase. A further centrifugation sedimented the proteins, the supernatant was removed and the pellet was dried in a vacuum centrifuge.

For one-dimensional separation of proteins, the pellets were dissolved in 10% glycerol, 2.3% SDS, 5% β-mercaptoethanol, 0.25% bromphenol blue, 63 mM Tris-HCl (pH 6.8). The 2-D Quant Kit (GE Healthcare, http://www.gehealthcare.com) was used for determining the protein concentration. A sample of 10 μg was separated by SDS-PAGE according to Laemmli [45]. The two-dimensional separation of proteins was accomplished as described in Zörb *et al*. [46] with the following modifications. Protein pellets were first dissolved in 8 M urea, 2 M thiourea, 0.5% IPG (immobilized pH gradient) buffer, 4% w/v CHAPS, 30 mM DTT, 20 mM Tris and then dialyzed using 3.5 kDa cut-off membrane (ZelluTrans, Carl Roth, http://www.carlroth.com) against the same buffer. The protein concentration was determined with the 2-D Quant Kit (GE Healthcare) and 25 μg of protein were separated on IPG strips of 7 cm in length with pH gradient of 3-10. Protein gels were stained according to the hot-staining protocol with Coomassie R350 tablets (PlusOne Coomassie tablets PhastGel Blue R-350, GE Healthcare) [47] and digitized with an Epson Perfection V700 Photo scanner (Epson, http://www.epson.com).

### LC-MS-based protein identification

Dialyzed protein extracts were precipitated by chloroform/methanol method and about 30 μg of protein were resolubilized in 50 μl 0.1% Rapigest (Waters Corporation, http://www.waters.com) in 50 mM ammonium bicarbonate. Protein concentrations were determined using the Bradford method [48] and bovine serum albumin as standard protein. Five μg of protein were reduced, alkylated and digested with trypsin over night at 37°C as described earlier [49]. The enzymatic reaction was stopped with 1N HCl and peptide solutions were adjusted to 0.1 μg/μl final concentration.

Three μl of protein digest were used for LC-separation on a nanoAcquity UPLC system (Waters) followed by mass spectrometry analysis on a Q-TOF Premier MS instrument (Waters) in a data directed analysis (DDA) mode, as described in Agrawal et al. [50].

Peptide separation was performed on a 180 μm × 20 mm Symmetry (5 μm) C18 precolumn (Waters) coupled to a 150 mm × 75 μm BEH130 (1.7 μm) C18 column (Waters), with a gradient of 3-40% actonitrile over 90 min. The MS operated in a positive ion mode with a source temperature of 80°C, a cone gas flow of 50 l/h, and a capillary voltage of approximately 3 kV. Mass spectra were acquired in a continuum V-mode and spectra integrated over 1 s intervals using MassLynx 4.1 software (Waters). The instrument was calibrated using selected fragment ions of the CID (collision-induced dissociation) of Glu-Fibrinopeptide B (SIGMA-ALDRICH, http://www.sigmaaldrich.com). Automatic data directed analysis (DDA) was employed for MS/MS analysis on doubly and triply charged precursor ions. The MS spectra were collected from *m/z *400 to *m/z *1600, and product ion MS/MS spectra were collected from *m/z *50 to *m/z *1600. Lock mass correction of the precursor and product ions was conducted with 500 fmol/μl Glu-Fibrinopeptide B in 0.1% formic acid in AcN/water (50:50, v/v) respectively, and introduced via the reference sprayer of the NanoLockSpray interface. ProteinLynx GlobalSERVER v2.3 software was used as a software platform for data processing, deconvolution, de novo sequence annotation of the spectra, and database search. A 10 ppm peptide, 0.1 Da fragment tolerance, one missed cleavage, and variable oxidation (Met) and carbamidomethylation (Cys) were used as the search parameters.

The resulting mass spectra were searched against the protein index of the UniProt *viridiplantae *database (release: July 2010 with 722.718 protein sequences) for protein identification applying the algorithm implemented in the ProteinLynxGlobalServer software (PLGS, Waters Cooperation). All samples were run as technical triplicates. Protein identifications consistent in two out of three LC-MS runs were considered as present in that sample. The false discovery rate was set to 4% of proteins included in the database.

Hierarchical clustering of protein abundances was performed using Gene Expression Similarity Investigation Suite Genesis v1.7.6 [51]. Average linkage clustering was applied for LC-MS experiments and protein abundances.

## Abbreviations

LC-MS/MS: liquid chromatography tandem mass spectrometry; 1-DE: one-dimensional gel electrophoresis; 2-DE: two-dimensional gel electrophoresis

## Competing interests

The authors declare that they have no competing interests.

## Authors' contributions

KW carried out protein extractions, protein separations, data evaluation and manuscript preparation. MS performed plant cultivation, apoplastic preparations and contributed to protein separations. AM conceived mass spectrometry analyses and contributed to manuscript writing. HPM participated in discussions during experimental work and manuscript preparation. KHM conceived the project and worked on manuscript preparation. All authors read and approved the final manuscript.

## Supplementary Material

Additional file 1 **Biological reproducibility of protein profiles from the maize leaf apoplast as resolved by 1-DE**. Apoplastic proteins were extracted with water (i) or 100 mM sodium phosphate buffer (ii). Two independent experiments were performed to assure consistent protein patterns. Click here for file

Additional file 2 **Identification of proteins from the apoplast of maize leaves**. UniProt database identifiers, along with molecular weight (mW) and isoelectric point (pI) are shown. The cellular localisation was assigned using Expasy tools Target P (http://www.cbs.dtu.dk/services/TargetP/) and WoLF PSORT (http://wolfpsort.org/). The identification of the respective proteins using different extraction solutions is indicated (i: water, ii: 100 mM sodium phosphate buffer, iii: 25 mM Tris-HCl, iv: 100 mM sorbitol, v: 20 mM ascorbic acid/20 mM CaCl_2_, vi: 50 mM NaCl). Click here for file

Additional file 3 **Identification of proteins from the apoplast of maize leaves**. Provided are the UniProt database identifiers, the PLGS score, probability score for identification, number of identified peptides, protein coverage and the peptide sequence. Click here for file

Additional file 4 **Proteins identified in apoplast extracts of all six infiltration solutions**. Click here for file

Additional file 5 **Example of protein identification from apoplastic extracts using nanoLC-ESI-Q-TOF MS**. The database search against the protein index of UniProt led to the identification of exhydrolase II [Q9XE93]. The amino acid sequence of the corresponding protein is shown on top with the detected peptides underlined. The *de novo *sequence of a selected peptide with precursor mass *m/z *859.4698 (charge 3) is shown. This peptide is marked in bold within the protein sequence. Click here for file
