# Supplementary figures and images for: Comparative evaluation of extraction methods for apoplastic proteins from maize leaves
Source: Plant Methods. 2011 Dec 22;7:48. doi: 10.1186/1746-4811-7-48 (PMC3284877; doi:10.1186/1746-4811-7-48)

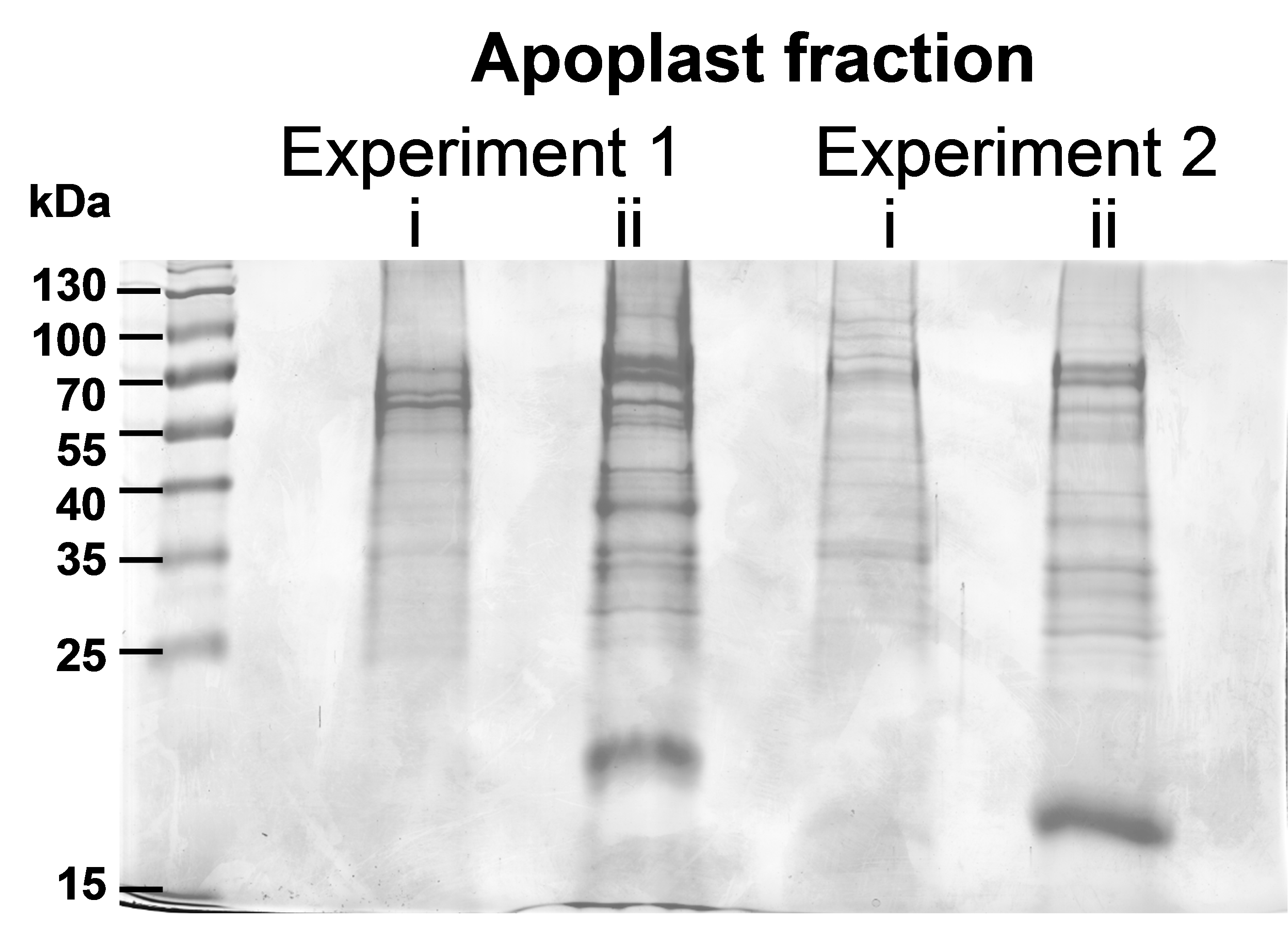

Supplement: Additional file 1 — Biological reproducibility of protein profiles from the maize leaf apoplast as resolved by 1-DE. Apoplastic proteins were extracted with water (i) or 100 mM sodium phosphate buffer (ii). Two independent experiments were performed to assure consistent protein patterns. [file 1746-4811-7-48-S1.DOC]

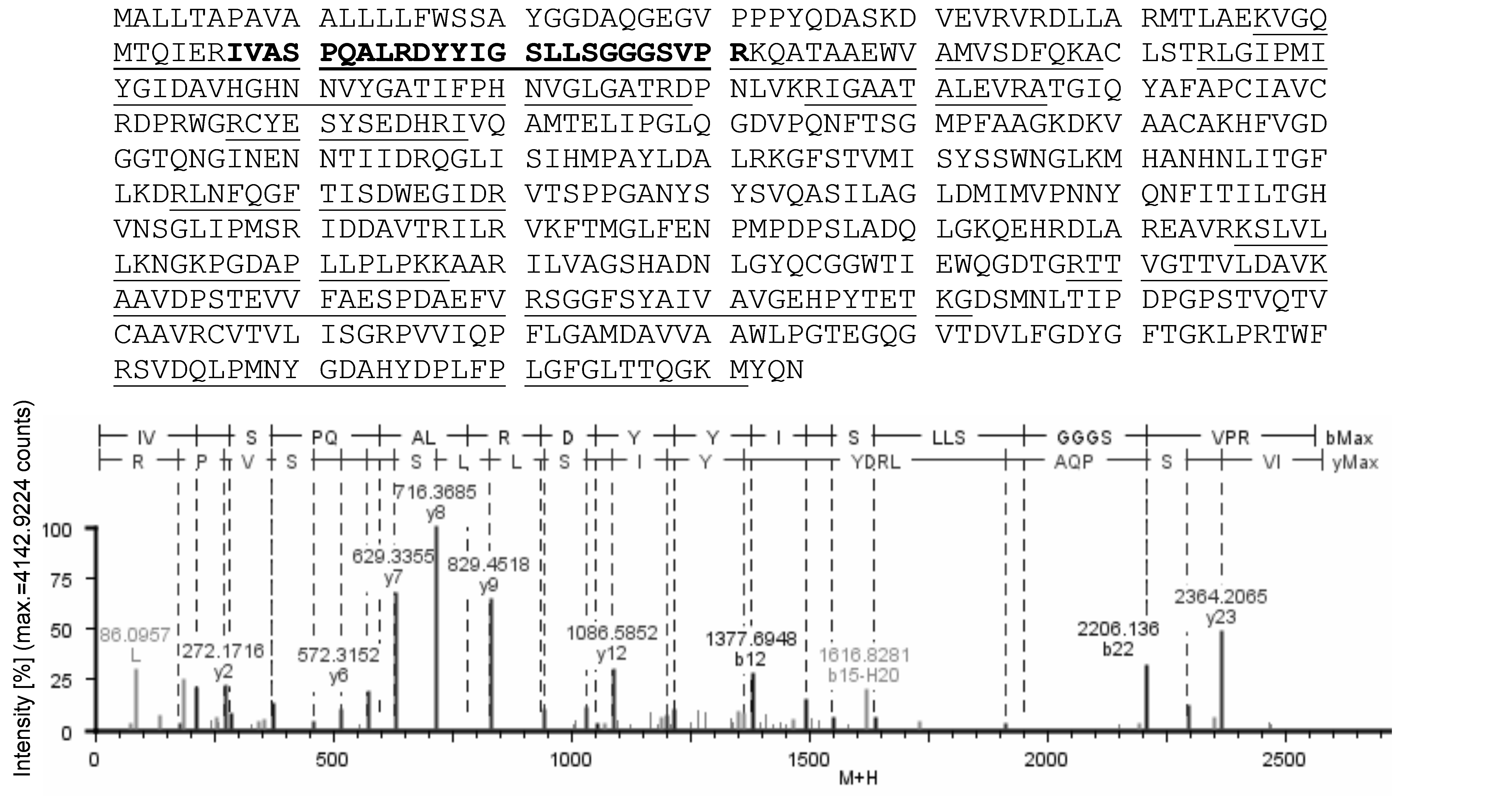

Supplement: Additional file 5 — Example of protein identification from apoplastic extracts using nanoLC-ESI-Q-TOF MS. The database search against the protein index of UniProt led to the identification of exhydrolase II [Q9XE93]. The amino acid sequence of the corresponding protein is shown on top with the detected peptides underlined. The de novo sequence of a selected peptide with precursor mass m/z 859.4698 (charge 3) is shown. This peptide is marked in bold within the protein sequence. [file 1746-4811-7-48-S5.DOC]
