# Supplementary material for: Comparative evaluation of extraction methods for apoplastic proteins from maize leaves
Source: Plant Methods. 2011 Dec 22;7:48. doi: 10.1186/1746-4811-7-48 (PMC3284877; doi:10.1186/1746-4811-7-48)
Supplement: Additional file 2 — Identification of proteins from the apoplast of maize leaves. UniProt database identifiers, along with molecular weight (mW) and isoelectric point (pI) are shown. The cellular localisation was assigned using Expasy tools Target P (http://www.cbs.dtu.dk/services/TargetP/) and WoLF PSORT (http://wolfpsort.org/). The identification of the respective proteins using different extraction solutions is indicated (i: water, ii: 100 mM sodium phosphate buffer, iii: 25 mM Tris-HCl, iv: 100 mM sorbitol, v: 20 mM ascorbic acid/20 mM CaCl2, vi: 50 mM NaCl). [file 1746-4811-7-48-S2.DOC]

| **Entry** | **Description** | **mW (Da)** | **pI (pH)** | **Localisation** | **Infiltration solutions** | | | | | |
| --- | --- | --- | --- | --- | --- | --- | --- | --- | --- | --- |
|  |  |  |  |  | **i** | **ii** | **iii** | **iv** | **v** | **vi** |
| **Metabolism** | | | | | | | | | | |
| UniRef90_B4FRC8 | Fruit protein, Zea mays | 31586 | 7.04 | chloroplast | x |  |  |  | x |  |
| UniRef90_B6SQQ0 | Inorganic pyrophosphatase, Zea maize | 31717 | 5.69 | cytosol | x | x | x | x |  | x |
| UniRef90_B6TVG5 | Arginine decarboxylase, Zea mays | 70655 | 5.02 | cytosol | x |  |  |  |  |  |
| UniRef90_B9HQI3 | Vitamin b12 independent methionine synthase, Populus trichocarpa | 84519 | 6.15 | cytosol | x |  |  |  |  |  |
| UniRef90_B9VU15 | Malic enzyme, Echinochloa crus | 55823 | 7.62 | chloroplast |  |  |  | x | x |  |
| UniRef90_C8ZJX1 | Malic enzyme, Aristida rhiniochloa | 40044 | 5.29 | chloroplast |  | x | x | x | x |  |
| UniRef90_P08170 | Seed lipoxygenase 1, Glycine max | 94310 | 5.93 | cytosol | x |  |  |  |  |  |
| UniRef90_P09186 | Seed lipoxygenase 3, Glycine max | 96697 | 6.25 | cytosol | x |  |  |  |  |  |
| UniRef90_P16243 | Malic enzyme, Zea mays | 69779 | 6.05 | chloroplast | x | x |  | x | x | x |
| UniRef90_P17847 | Ferredoxin nitrite reductase, Zea maize | 63301 | 6.00 | chloroplast |  | x |  |  |  |  |
| UniRef90_P29530 | P24 oleosin isoform A, Glycine max | 23487 | 9.05 | vacuole | x |  |  |  |  |  |
| UniRef90_Q42662 | 5-methyltetrahydropteroyltriglutamate homocysteine methyltransferase, Solenostemon scutellar | 84536 | 6.06 | cytosol | x | x |  | x |  |  |
| UniRef90_Q4H1G2 | Methionine synthase, Beta vulgaris | 87747 | 6.01 | cytosol | x |  |  |  |  |  |
| UniRef90_Q56WQ1 | Aspartate aminotransferase, Arabidopsis thaliana | 20685 | 8.69 | chloroplast | x | x | x |  | x |  |
| **Energy** | | | | | | | | | | |
| UniRef90_A3C0Z2 | Triosephosphate isomerase, Oryza sativa | 25993 | 8.70 | chloroplast |  | x |  |  | x | x |
| UniRef90_A5BDH7 | Fructose bisphosphate aldolase, Vitis vinifera | 42921 | 8.07 | chloroplast | x |  |  | x | x |  |
| UniRef90_A6YF91 | Ribulose bisphosphate carboxylase oxygenase large subunit, uncultured eukaryote | 23991 | 9.63 | cytosol | x |  | x | x |  |  |
| UniRef90_A7DX17 | Phosphoenolpyruvate carboxylase, Cyrtococcum patens | 70953 | 6.19 | cytosol | x |  | x | x |  |  |
| UniRef90_A7DX26 | Phosphoenolpyruvate carboxylase, Streptochaeta sodiroana | 49904 | 5.34 | cytosol | x |  | x | x | x | x |
| UniRef90_A7DX31 | Phosphoenolpyruvate carboxylase, Brachypodium pinnatum | 49559 | 5.40 | cytosol |  |  |  |  |  | x |
| UniRef90_A7DX42 | Phosphoenolpyruvate carboxylase, Dactyloctenium aegyptium | 49540 | 6.93 | cytosol | x |  | x | x | x | x |
| UniRef90_A7DX45 | Phosphoenolpyruvate carboxylase, Digitaria didactyla | 49403 | 5.72 | cytosol | x |  | x | x | x | x |
| UniRef90_A7DX70 | Phosphoenolpyruvate carboxylase, Melinis minutiflora | 49628 | 6.68 | cytosol | x |  | x | x | x | x |
| UniRef90_A7DXA5 | Phosphoenolpyruvate carboxylase, Panicum capillare | 49803 | 6.47 | cytosol | x | x | x | x | x | x |
| UniRef90_A7DXE1 | Phosphoenolpyruvate carboxylase, Tristachya leucothrix | 49613 | 6.35 | cytosol | x | x | x | x | x | x |
| UniRef90_A8ASG2 | Phosphoenolpyruvate carboxylase, Aloe arborescens | 110128 | 6.02 | cytosol | x | x | x | x | x | x |
| UniRef90_B0B714 | Ribulose bisphosphate carboxylase large chain, Sorghum bicolor | 49380 | 6.45 | cytosol |  |  |  | x |  |  |
| UniRef90_B0LXE5 | Phosphoenolpyruvate carboxylase, Arachis hypogaea | 110719 | 5.91 | cytosol | x |  | x | x | x |  |
| UniRef90_B3SRP7 | PsbP, Phyllostachys edulis | 12603 | 4.41 | chloroplast | x |  |  |  |  |  |
| UniRef90_B4FCE2 | Triosephosphate isomerase, Zea mays | 30580 | 7.13 | chloroplast | x |  | x | x |  |  |
| UniRef90_B5SW42 | Ribulose bisphosphate carboxylase large chain, Karroochloa schismoides | 36660 | 7.36 | cytosol |  | x |  |  |  | x |
| UniRef90_B6SKI1 | Photosystem I reaction center subunit II, Zea mays | 21974 | 10.12 | chloroplast | x |  |  | x |  |  |
| UniRef90_B6SSB9 | Plastocyanin, Zea mays | 15860 | 5.53 | chloroplast | x |  | x | x | x | x |
| UniRef90_B6SSU6 | Fructose bisphosphate aldolase, Zea maize | 38178 | 6.24 | cytosol |  |  | x | x | x |  |
| UniRef90_B6SUC4 | Chlorophyll a b binding protein 8, Zea maize | 28965 | 9.31 | chloroplast |  |  |  | x |  |  |
| UniRef90_B6T9S5 | Ferredoxin NADP reductase, Zea mays | 40634 | 8.43 | chloroplast | x |  | x | x | x | x |
| UniRef90_B6TEW2 | Ferredoxin NADP reductase, Zea mays | 37593 | 8.32 | chloroplast | x | x | x | x | x | x |
| UniRef90_B7ZYT6 | Ribulose bisphosphate carboxylase small chain, Zea mays | 17847 | 7.99 | cytosol |  |  | x | x |  |  |
| UniRef90_B9HFI4 | Fructose bisphosphate aldolase, Populus trichocarpa | 42723 | 7.03 | chloroplast | x |  |  | x |  |  |
| UniRef90_C1DZM5 | Phosphoenolpyruvate carboxylase, Micromonas sp. | 110457 | 5.59 | cytosol | x |  | x |  | x |  |
| UniRef90_C8CIR2 | Malate dehydrogenase, Camellia sinensis | 15193 | 7.99 | cytosol | x |  | x |  |  |  |
| UniRef90_D2T2H9 | Phosphoenolpyruvate carboxylase, Holcus lanatus | 56843 | 6.27 | cytosol | x | x | x | x | x | x |
| UniRef90_D3KY86 | Phosphoenolpyruvate carboxylase, Ananas comosus | 112518 | 5.77 | cytosol | x | x | x | x | x | x |
| UniRef90_O22387 | Glyceraldehyde 3-phosphate dehydrogenase, Oryza sativa | 28313 | 9.98 | cytosol |  |  |  |  | x | x |
| UniRef90_O22534 | Glyceralehyde 3-phosphate dehydrogenase, Oryza sativa | 36421 | 9.89 | chloroplast | x | x |  |  |  |  |
| UniRef90_P00291 | Plastocyanin, Sambucus nigra | 10454 | 4.08 | chloroplast |  |  | x | x | x | x |
| UniRef90_P05348 | Ribulose bisphosphate carboxylase small chain, Zea mays | 19138 | 9.10 | chloroplast | x |  | x |  | x |  |
| UniRef90_P11155 | Pyruvate phosphate dikinase 1, Zea maize | 102609 | 5.66 | chloroplast | x | x | x | x | x | x |
| UniRef90_P15804 | Phosphoenolpyruvate carboxylase 3 , Sorghum bicolor | 108304 | 5.85 | cytosol | x |  |  |  |  |  |
| UniRef90_P15804 | Phosphoenolpyruvate carboxylase 3, Sorghum bicolor | 108304 | 5.85 | cytosol |  | x | x | x | x | x |
| UniRef90_P20120 | Photosystem I reaction center subunit V, Pisum sativum | 4394 | 7.18 | chloroplast |  |  |  | x |  |  |
| UniRef90_P25858 | Glyceraldehyde 3-phosphate dehydrogenase, Arabidopsis thaliana | 36891 | 6.72 | cytosol | x |  |  | x |  |  |
| UniRef90_P26667 | Ribulose bisphosphate carboxylase small chain, Triticum aestivum | 19441 | 8.27 | chloroplast |  |  |  | x |  |  |
| UniRef90_P26958 | Ribulose bisphosphate carboxylase large chain, Bryopsis maxima | 52597 | 6.37 | cytosol | x |  |  | x |  |  |
| UniRef90_P26985 | Ribulose bisphosphate carboxylase small chain, Batophora oerstedii | 20096 | 7.63 | chloroplast |  |  |  | x |  |  |
| UniRef90_P29195 | Phosphoenolpyruvate carboxylase 1, Sorghum bicolor | 109370 | 5.67 | cytosol | x |  |  | x | x |  |
| UniRef90_P48497 | Triosephosphate isomerase, Stellaria longipes | 27482 | 5.40 | cytosol |  |  | x | x |  |  |
| UniRef90_P51063 | Phosphoenolpyruvate carboxylase, Picea abies | 109483 | 5.90 | cytosol |  |  |  |  | x |  |
| UniRef90_P93696 | Phosphoenolpyruvate carboxylase, Vanilla planifolia | 109212 | 6.04 | cytosol | x |  | x |  | x |  |
| UniRef90_Q05800 | Ribulose bisphosphate carboxylase large chain, Nelumbo lutea | 43889 | 6.42 | cytosol | x |  | x |  |  |  |
| UniRef90_Q10HD0 | Chlorophyll a b binding protein, Oryza sativa | 28477 | 5.52 | chloroplast |  |  | x | x |  | x |
| UniRef90_Q10SA2 | Glyceraldehyde 3-phosphate dehydrogenase B, Oryza sativa | 33682 | 4.81 | chloroplast |  |  | x | x | x | x |
| UniRef90_Q198V8 | Phosphoenolpyruvate carboxylase, Suaeda eltonica | 94407 | 6.93 | cytosol | x | x | x | x | x | x |
| UniRef90_Q1RS11 | Malate dehydrogenase, Paspalidium geminatum | 39298 | 4.92 | cytosol | x |  | x |  | x | x |
| UniRef90_Q1WFH4 | Phosphoenolpyruvate carboxylase, Clusia minor | 41292 | 8.93 | cytosol |  |  |  | x | x |  |
| UniRef90_Q1XAT9 | Phosphoenolpyruvate carboxylase, Alternanthera pungens | 109809 | 6.25 | cytosol | x |  | x | x | x |  |
| UniRef90_Q20FB3 | Glyceraldehyde 3-phosphate dehydrogenase subunit B, Spirogyra sp. | 34198 | 5.97 | cytosol | x |  |  |  |  |  |
| UniRef90_Q20FC2 | Glyceraldehyde 3-phosphate dehydrogenase subunit A, Cladophora rupestris | 40926 | 9.29 | cytosol | x |  |  | x |  |  |
| UniRef90_Q2RA02 | Fructose bisphosphate aldolase, Oryza sativa | 25186 | 6.38 | cytosol |  | x |  |  |  |  |
| UniRef90_Q38768 | 3-phosphoglycerate kinase, Avena sativa | 9402 | 7.62 | cytosol |  |  |  | x |  |  |
| UniRef90_Q3LR52 | Phosphoenolpyruvate carboxylase, Zea mays | 20292 | 5.13 | cytosol | x |  | x | x | x | x |
| UniRef90_Q40677 | Fructose bisphosphate aldolase, Oryza sativa | 41979 | 6.42 | chloroplast | x |  |  | x |  |  |
| UniRef90_Q41746 | Chlorophyll a b binding apoprotein CP26, Zea maize | 30151 | 5.36 | chloroplast |  |  |  | x |  |  |
| UniRef90_Q43831 | Ribulose bisphosphate carboxylase large subunit binding protein subunit beta, Secale cereale | 53379 | 4.68 | chloroplast | x |  |  | x |  |  |
| UniRef90_Q52NW0 | Phosphoenolpyruvate carboxylase, Echinochloa crus | 109316 | 5.60 | cytosol | x | x | x | x | x | x |
| UniRef90_Q53P93 | Fructose bisphosphate aldolase, Oryza sativa | 15834 | 7.16 | cytosol |  |  |  |  |  | x |
| UniRef90_Q53P96 | Fructose bisphosphate aldolase, Oryza sativa | 39328 | 7.11 | cytosol |  |  | x |  |  |  |
| UniRef90_Q5D5T4 | Phosphoenolpyruvate carboxylase, Hordeum spontaneum | 55721 | 5.36 | cytosol | x |  | x |  |  |  |
| UniRef90_Q6AVA8 | Pyruvate phosphate dikinase 1, Oryza sativa | 102722 | 5.93 | chloroplast |  | x |  |  |  |  |
| UniRef90_Q6LBU9 | Glyceraldehyde 3-phosphate dehydrogenase, Zea maize | 40923 | 7.17 | cytosol |  |  |  | x |  |  |
| UniRef90_Q8H959 | Phosphoenolpyruvate carboxylase, Eleocharis vivipara | 110738 | 5.93 | cytosol |  |  |  |  | x |  |
| UniRef90_Q8RVZ5 | Phosphoenolpyruvate carboxylase, Vetiveria zizanioides | 45483 | 5.19 | cytosol | x |  | x | x | x | x |
| UniRef90_Q8VX35 | Phosphoenolpyruvate carboxylase, Vanilla planifolia | 41090 | 9.25 | cytosol | x |  | x | x | x |  |
| UniRef90_Q8VXE5 | Phosphoenolpyruvate carboxylase, Mesembryanthemum crystallinum | 41344 | 9.82 | cytosol | x |  | x | x |  |  |
| UniRef90_Q8WL30 | Ribulose bisphosphate carboxylase large chain, Hydnocarpus heterophyllus | 51379 | 5.97 | cytosol |  |  |  | x |  |  |
| UniRef90_Q93XG7 | Phosphoenolpyruvate carboxylase 3, Hydrilla verticillata | 110270 | 5.80 | cytosol | x |  | x | x | x | x |
| UniRef90_Q944G9 | Fructose bisphosphate aldolase 2, Arabidopsis thaliana | 42915 | 7.18 | cytosol | x |  |  | x |  |  |
| UniRef90_Q946P2 | Ribulose bisphosphate carboxylase oxygenase large subunit, uncultured Chlorophyta | 21827 | 7.35 | cytosol | x | x | x | x |  | x |
| UniRef90_Q9BAH7 | Ribulose bisphosphate carboxylase large chain, Agathis robusta | 51747 | 6.22 | cytosol |  |  |  | x |  |  |
| UniRef90_Q9FQ80 | Phosphoenolpyruvate carboxylase, Chloris gayana | 108449 | 5.68 | cytosol | x |  | x |  | x |  |
| UniRef90_Q9FV16 | Glyceraldehyde 3-phosphate dehydrogenase B subunit, Cucurbita pepo | 18164 | 6.10 | chloroplast | x |  | x | x |  | x |
| UniRef90_Q9SLP6 | Ferredoxin NADP reductase, Zea maize | 39297 | 8.42 | chloroplast |  |  |  |  |  | x |
| UniRef90_Q9SNY6 | Pyruvate orthophosphate dikinase, Saccharum officinarum | 102470 | 5.39 | cytosol |  | x |  |  |  |  |
| **Cell growth and division** | | | | | | | | | | |
| UniRef90_B9SBU7 | Dynamin 2A, Ricinus communis | 90140 | 9.20 | nucleus |  |  |  | x |  |  |
| UniRef90_P43188 | Adenylate kinase, Zea mays | 24851 | 4.75 | chloroplast | x |  | x | x |  |  |
| UniRef90_Q2R2F0 | AT hook motif family protein, Oryza sativa | 43917 | 8.52 | nucleus |  | x |  |  | x |  |
| UniRef90_Q6ZDE3 | Abscisic acid 8'-hydroxylase 2, Oryza sativa | 56519 | 9.83 | extracellular | x |  |  |  |  |  |
| UniRef90_Q9LVN7 | DNA polymerase delta catalytic subunit, Arabidopsis thaliana | 121659 | 8.61 | nucleus | x |  |  |  |  |  |
| **Transcription** | | | | | | | | | | |
| UniRef90_B6TK28 | Myb-like DNA-binding protein, Zea maize | 36712 | 7.18 | nucleus | x |  |  |  |  |  |
| UniRef90_B9RVT3 | Homeobox protein, Ricinus communis | 199727 | 5.31 | nucleus |  |  |  | x |  |  |
| **Protein destination and storage** | | | | | | | | | | |
| UniRef90_A4PIZ1 | Cysteine proteinase, Lotus japonicus | 37644 | 4.68 | chloroplast |  |  |  |  | x |  |
| UniRef90_A4PIZ3 | Cysteine proteinase, Lotus japonicus | 37384 | 5.79 | chloroplast | x |  | x |  |  |  |
| UniRef90_A5WYF8 | Seed storage protein A, Vigna luteola | 49839 | 4.91 | vacuole | x |  |  |  |  |  |
| UniRef90_B6SVE1 | Serine carboxypeptidase 1, Zea mays | 56423 | 5.36 | cytosol |  |  |  |  |  | x |
| UniRef90_B6TDA5 | Serine carboxypeptidase 1, Zea mays | 56065 | 5.55 | cytosol | x | x | x |  | x | x |
| UniRef90_B6TG95 | Vignain, Zea mays | 38823 | 4.68 | extracellular | x | x | x | x | x | x |
| UniRef90_B6TYX7 | Polygalacturonase inhibitor 1, Zea mays | 30011 | 8.08 | extracellular |  | x |  |  | x | x |
| UniRef90_B6U1P8 | Subtilisin-like protease, Zea mays | 77910 | 6.49 | vacuole | x | x | x |  |  | x |
| UniRef90_B6U5I1 | Peptidyl-prolyl-cis-trans-isomerase, Zea maize | 46610 | 4.71 | cytosol |  |  |  |  |  |  |
| UniRef90_B6U5I1 | Peptidyl-prolyl-cis-trans-isomerase, Zea maize | 46610 | 4.71 | cytosol |  |  |  |  | x | x |
| UniRef90_P02856 | Vicilin, Pisum sativum | 14031 | 5.08 | vacuole | x |  |  |  |  |  |
| UniRef90_P02858 | Glycinin B3 subunit, Glycine max | 63548 | 5.14 | vacuole | x |  |  |  |  |  |
| UniRef90_P04405 | Glycinin B1a subunit, Glycine max | 54356 | 5.30 | vacuole | x |  |  |  |  |  |
| UniRef90_P04776 | Glycinin Bx subunit, Glycine max | 55671 | 5.83 | vacuole | x |  |  |  |  |  |
| UniRef90_P11828 | Glycinin B subunit, Glycine max | 54207 | 5.63 | vacuole | x |  |  |  |  |  |
| UniRef90_P13916 | Beta conglycinin alpha chain, Glycine max | 70250 | 4.88 | vacuole | x |  |  | x |  |  |
| UniRef90_P19594 | 2S albumin large chain, Glycine max | 18447 | 5.02 | vacuole | x |  |  |  |  |  |
| UniRef90_P25974 | Beta conglycinin beta chain, Glycine max | 50521 | 5.82 | vacuole | x |  |  |  |  |  |
| UniRef90_Q01527 | Maturation protein, Glycine max | 15571 | 10.09 | vacuole | x |  |  |  |  |  |
| UniRef90_Q03971 | Legumin A1, Vicia faba | 56758 | 6.05 | vacuole | x |  |  |  |  |  |
| UniRef90_Q1WAB8 | Glycinin, Glycine microphylla | 63082 | 5.55 | vacuole | x |  |  |  |  |  |
| UniRef90_Q39858 | Soybean glycinin A3 B4 subunit, Glycine max | 27279 | 5.45 | vacuole | x |  |  |  |  |  |
| UniRef90_Q39871 | Late embryongenesis abundant protein, Glycine max | 50613 | 6.29 | vacuole | x |  |  |  |  |  |
| UniRef90_Q84UB3 | Beta conglycinin alpha subunit, Glycine max | 44991 | 5.49 | vacuole | x |  |  |  |  |  |
| UniRef90_Q8RVH5 | Basic 7S globulin, Glycine max | 47174 | 8.17 | vacuole | x |  |  |  |  |  |
| UniRef90_Q9ZNZ4 | Napin-type 2S albumin, Glycine max | 17822 | 5.96 | vacuole | x |  |  |  |  |  |
| **Transporters** | | | | | | | | | | |
| UniRef90_B4FB54 | Non-specific lipid transfer protein, Zea mays | 12084 | 9.60 | extracellular |  | x |  |  |  |  |
| UniRef90_B6SP11 | Non-specific lipid transfer protein, Zea mays | 9802 | 8.73 | extracellular |  | x |  |  |  |  |
| UniRef90_B6SY96 | Non-specific lipid transfer protein, Zea mays | 12011 | 9.29 | extracellular |  | x |  |  |  |  |
| UniRef90_B6TRB2 | Copper ion binding protein, Zea maize | 17057 | 9.78 | extracellular |  |  | x |  |  |  |
| UniRef90_P05046 | Lectin, Glycine max | 30908 | 5.60 | extracellular | x |  |  | x |  |  |
| UniRef90_P12085 | ATP synthase subunit beta, Oryza sativa | 53921 | 5.21 | chloroplast |  |  |  | x |  |  |
| UniRef90_P19656 | Non-specific lipid transfer protein, Zea mays | 11697 | 8.74 | extracellular |  | x |  |  |  | x |
| UniRef90_Q00X11 | ATPase type 13A, Ostreococcus tauri | 153915 | 6.87 | chloroplast |  | x |  |  |  |  |
| UniRef90_Q04672 | Sucrose-binding protein, Glycine max | 60484 | 6.42 | extracellular | x |  |  |  |  |  |
| UniRef90_Q8HDD9 | ATP synthase subunit beta, Dunaliella parva | 40752 | 5.14 | chloroplast | x |  |  | x |  | x |
| UniRef90_Q8S6Z1 | ATPase alpha subunit 3, Oryza sativa | 29298 | 5.10 | chloroplast | x |  |  | x |  |  |
| **Cell structure** | | | | | | | | | | |
| UniRef90_A5H454 | Peroxidase 66, Zea m aize | 33398 | 8.02 | extracellular |  |  |  |  | x |  |
| UniRef90_A5H8G4 | Peroxidase 1, Zea maize | 38330 | 6.89 | extracellular |  |  |  |  | x |  |
| UniRef90_A5JTQ2 | Alpha N arabinofuranosidase, Medicago varia | 83673 | 6.22 | extracellular |  | x |  |  |  |  |
| UniRef90_B4FKV6 | Peroxidase 54, Zea maize | 36178 | 4.95 | extracellular |  |  |  |  |  | x |
| UniRef90_B5AK47 | Dhurrinase-like B glucosidase, Zea maize | 64233 | 7.95 | extracellular |  |  | x |  |  |  |
| UniRef90_B6SMR2 | Peroxidase 52, Zea mays | 33504 | 8.14 | extracellular |  | x | x |  | x | x |
| UniRef90_B6SUH6 | Non-cyanogenic beta glucosidase, Zea mays | 56680 | 5.39 | extracellular | x |  | x |  | x |  |
| UniRef90_B6SWK9 | Auxin-induced beta glucosidase, Zea mays | 69541 | 8.22 | mitochondria |  |  |  | x |  |  |
| UniRef90_B6SXU7 | Heparanase-like protein 3, Zea maize | 58262 | 9.35 | extracellular | x |  | x | x | x | x |
| UniRef90_B6SXY3 | Beta galactosidase, Zea mays | 48728 | 8.38 | extracellular |  | x |  |  |  |  |
| UniRef90_B6T391 | Lichenase 2, Zea mays | 34951 | 5.64 | extracellular | x | x | x | x | x | x |
| UniRef90_B6T9B9 | Alpha N arabinofuranosidase, Zea mays | 74827 | 5.04 | extracellular |  |  | x |  | x | x |
| UniRef90_B6TU39 | Peroxidase 2, Zea maize | 34941 | 4.67 | extracellular |  | x |  |  |  |  |
| UniRef90_B6TU78 | Glucan endo-1-3 beta glucosidase 7, Zea maize | 45316 | 5.54 | extracellular | x |  |  |  |  |  |
| UniRef90_B6TXJ8 | Glycoside hydrolase family 28, Zea maize | 47066 | 5.58 | extracellular | x |  |  |  |  |  |
| UniRef90_B6U063 | Carboxylic ester hydrolase, Zea mays | 50032 | 7.85 | extracellular | x |  | x |  | x |  |
| UniRef90_B6U0W2 | Beta galactosidase, Zea maize | 93935 | 6.48 | extracellular |  | x |  |  |  |  |
| UniRef90_B9SD68 | Hydrolase, Ricinus communis | 67825 | 6.80 | extracellular | x | x | x |  |  |  |
| UniRef90_C4N559 | Xyloglucan endotransglycosylase hydrolase, Musa acuminata | 20309 | 9.54 | extracellular |  |  |  |  |  | x |
| UniRef90_C5WQU7 | Beta galactosidase, Sorghum bicolor | 92893 | 5.31 | extracellular | x |  | x | x | x | x |
| UniRef90_C5Z534 | Beta galactosidase, Sorghum bicolor | 79098 | 7.74 | extracellular |  | x |  |  |  |  |
| UniRef90_O04943 | Alpha galactosidase, Hordeum vulgare | 17730 | 6.33 | extracellular |  | x | x |  | x | x |
| UniRef90_P93518 | PRm 3, Zea maize | 30099 | 3.90 | extracellular | x | x | x | x | x | x |
| UniRef90_Q018N1 | Tubulin-like protein, Ostreococcus tauri | 59870 | 4.82 | cytosol |  |  |  |  | x |  |
| UniRef90_Q10CU3 | Glycosyl hydrolase family 3, Oryza sativa | 43916 | 8.20 | extracellular | x |  | x |  |  |  |
| UniRef90_Q10M79 | Alpha L arabinofuranosidase, Oryza sativa | 73965 | 4.73 | extracellular |  |  |  |  | x |  |
| UniRef90_Q10NX8 | Beta galactosidase 6, Oryza sativa | 92780 | 5.52 | extracellular | x |  |  | x |  |  |
| UniRef90_Q1EMQ3 | Profilin, Plantago major | 13915 | 4.49 | cytosol |  | x |  |  | x | x |
| UniRef90_Q2R3E0 | Glycosyl hydrolases family 38, Oryza sativa | 114085 | 5.85 | extracellular |  | x |  |  |  | x |
| UniRef90_Q2RAZ2 | Alpha L arabinofuranosidase, Oryza sativa | 73421 | 4.57 | extracellular | x |  | x | x | x | x |
| UniRef90_Q43417 | Peroxidase, Cenchrus ciliaris | 32473 | 7.50 | extracellular |  |  |  |  | x |  |
| UniRef90_Q53MP2 | Beta D-xylosidase, Oryza sativa | 82557 | 6.62 | extracellular |  | x |  |  |  |  |
| UniRef90_Q5CCP6 | Beta galactosidase, Pyrus pyrifolia | 94782 | 8.12 | extracellular |  | x |  |  |  |  |
| UniRef90_Q5I3F3 | Peroxidase 5, Triticum monococcum | 27533 | 5.72 | extracellular |  | x |  |  |  |  |
| UniRef90_Q5I7F9 | Actin, Botryosphaeria rhodina | 17966 | 5.02 | cytosol |  |  |  | x |  |  |
| UniRef90_Q6L619 | Beta galactosidase, Raphanus sativus | 92580 | 8.36 | extracellular | x |  |  |  | x |  |
| UniRef90_Q7G3T8 | Beta galactosidase 13, Oryza sativa | 91940 | 6.06 | extracellular |  | x |  |  |  |  |
| UniRef90_Q8GUY1 | Pectinesterase, Lolium perenne | 24837 | 7.81 | extracellular |  | x |  |  |  |  |
| UniRef90_Q8RUV9 | Beta galactosidase 1, Oryza sativa | 91652 | 5.71 | extracellular |  | x |  |  |  |  |
| UniRef90_Q9FXT4 | Alpha galactosidase, Oryza sativa | 45792 | 7.91 | extracellular |  |  |  |  | x | x |
| UniRef90_Q9LLB8 | Exoglucanase, Zea mays | 66900 | 6.99 | extracellular | x | x | x | x |  | x |
| UniRef90_Q9XE93 | Exhydrolase II, Zea mays | 68330 | 6.13 | extracellular | x | x | x | x | x | x |
| UniRef90_Q9XEI3 | Beta D-glucan exohydrolase isoenzyme, Hordeum vulgare | 67862 | 6.24 | extracellular | x | x |  | x |  |  |
| **Signal transduction** | | | | | | | | | | |
| UniRef90_B6TWC3 | Rhicadhesin receptor, Zea mays | 23726 | 9.20 | extracellular |  | x | x |  |  |  |
| UniRef90_B9MZ47 | Fasciclin-like AGP 14 4 protein, Populus trichocarpa | 24786 | 8.63 | extracellular |  |  |  |  | x |  |
| UniRef90_C3US93 | ARK3, Capsella rubella | 31328 | 4.64 | cytosol |  |  |  |  | x |  |
| **Disease and defence** | | | | | | | | | | |
| UniRef100_P13867 | Alpha amylase trypsin inhibitor, Zea mays | 22060 | 7.69 | extracellular |  | x | x |  | x | x |
| UniRef100_P25272 | Kunitz-type trypsin inhibitor 1, Glycine max | 22531 | 4.77 | extracellular | x |  |  |  |  |  |
| UniRef90_A7IZL3 | Invertase inhibitor, Coffea canephora | 20205 | 6.68 | extracellular |  | x |  |  |  |  |
| UniRef90_B3VQP6 | Asorbate peroxidase 1, Eleusine coracana | 5967 | 4.33 | cytosol | x |  |  |  |  |  |
| UniRef90_B6T9B3 | 2-cys peroxiredoxin, Zea mays | 28216 | 5.71 | cytosol | x |  | x | x | x | x |
| UniRef90_B6TA80 | Thaumatin-like protein, Zea mays | 17632 | 6.75 | extracellular |  |  |  |  | x |  |
| UniRef90_B6TDW7 | Secretory protein, Zea mays | 24467 | 4.64 | extracellular |  |  |  |  | x |  |
| UniRef90_B6TT00 | Endochitinase PR4, Zea maize | 28545 | 4.96 | extracellular | x |  |  |  | x |  |
| UniRef90_B6TTY1 | Germin-like protein, Zea maize | 26764 | 7.25 | extracellular | x |  | x |  | x | x |
| UniRef90_B6TWH6 | Lysosomal Pro X carboxypeptidase, Zea mays | 59936 | 5.62 | extracellular | x | x | x | x |  | x |
| UniRef90_B6UB57 | Lysosomal protective protein, Zea maize | 53540 | 5.87 | extracellular | x | x | x |  | x | x |
| UniRef90_B9N1I3 | Thioredoxin M, Populus trichocarpa | 13108 | 5.74 | chloroplast | x |  |  |  | x | x |
| UniRef90_O24007 | Chitinase, Oryza sativa | 18956 | 4.83 | extracellular | x |  | x | x | x | x |
| UniRef90_P01063 | Bowman-Birk-type proteinase inhibitor C II, Glycine max | 9194 | 4.38 | extracellular | x |  |  |  |  |  |
| UniRef90_P29022 | Endochitinase A, Zea maize | 29105 | 7.85 | extracellular |  |  |  |  | x |  |
| UniRef90_Q39805 | Dehydrin-like protein, Glycine max | 23703 | 6.08 | cytosol | x |  |  |  |  |  |
| UniRef90_Q41864 | Thioredoxin M, Zea maize | 18061 | 8.40 | chloroplast | x |  |  |  | x | x |
| UniRef90_Q5U1S9 | Class III peroxidase 14, Oryza sativa | 37174 | 5.77 | extracellular |  |  |  |  | x |  |
| UniRef90_Q6EUS1 | Class III peroxidase 27, Oryza sativa | 33300 | 8.09 | extracellular |  | x |  |  |  | x |
| UniRef90_Q6TM44 | Germin-like protein, Zea mays | 21873 | 6.04 | extracellular | x | x | x | x | x | x |
| UniRef90_Q7M1R1 | Chitinase, Gladiolus x gandavensis | 30695 | 5.76 | extracellular | x |  | x | x | x | x |
| **Secondary metabolism** | | | | | | | | | | |
| UniRef90_O64411 | Polyamine oxidase, Zea mays | 56308 | 5.63 | extracellular |  | x |  |  |  |  |
| **Unclear classification** | | | | | | | | | | |
| UniRef90_B6TUR7 | Blue copper protein, Zea mays | 18091 | 7.16 | not clear | x |  |  |  |  |  |
| UniRef90_Q9FXL2 | Vf14-3-3c protein, Vicia faba | 29664 | 4.59 | cytosol |  |  |  | x |  |  |
| **Unclassified** | | | | | | | | | | |
| UniRef90_A3CAN2 | Putative uncharacterized protein, Oryza sativa | 63839 | 6,02 | unknown |  | x |  |  |  |  |
| UniRef90_A5B0V7 | Putative uncharacterized protein, Vitis vinifera | 94461 | 8,66 | unknown |  | x |  |  |  |  |
| UniRef90_A9RZ84 | Predicted protein, Physcomitrella patens | 34447 | 8,78 | unknown |  | x |  |  |  |  |
| UniRef90_A9SBT7 | Predicted protein, Physcomitrella patens | 28880 | 11,18 | unknown |  | x |  |  |  |  |
| UniRef90_B4FQ31 | Putative uncharacterized protein, Zea mays | 48738 | 5,87 | unknown |  | x |  |  |  |  |
| UniRef90_B4FT96 | Putative uncharacterized protein, Zea mays | 17440 | 5,19 | unknown |  | x |  |  |  |  |
| UniRef90_B8BGH2 | Putative uncharacterized protein, Oryza sativa | 53620 | 7,38 | unknown |  | x |  |  |  |  |
| UniRef90_C0PLT8 | Putative uncharacterized protein, Zea mays | 58742 | 5,23 | unknown |  | x |  |  |  |  |
| UniRef90_C6JRI5 | Putative uncharacterized protein, Sorghum bicolor | 80282 | 6,40 | unknown |  | x |  |  |  |  |
| UniRef90_Q0J290 | Os09g0368500 protein, Oryza sativa | 53224 | 4,90 | unknown |  | x |  |  |  |  |
| UniRef90_Q6F2N2 | Putative glycoside hydrolase, Oryza sativa | 30869 | 5,24 | unknown |  | x |  |  |  |  |
| UniRef90_Q947C6 | Putative nodulin-like protein, Triticum monococcum | 43563 | 9,62 | unknown |  | x |  |  |  |  |
| UniRef90_A1Z263 | Putative vitamin B 12 independent methionine synthase, Pisum sativum | 34651 | 5,68 | unknown | x |  |  |  |  |  |
| UniRef90_A2Q1E1 | Putative uncharacterized protein, Medicago truncatula | 15204 | 7,35 | unknown |  |  |  |  |  | x |
| UniRef90_A2XJC7 | Putative uncharacterized protein, Oryza sativa | 47359 | 7,16 | unknown | x | x | x | x | x | x |
| UniRef90_A2ZG56 | Putative uncharacterized protein, Oryza sativa | 111459 | 6,35 | unknown |  |  |  | x | x |  |
| UniRef90_A3BCJ8 | Putative uncharacterized protein, Oryza sativa | 32219 | 6,95 | unknown |  |  |  |  |  | x |
| UniRef90_A4RUM7 | Predicted protein, Ostreococcus lucimarinus | 17652 | 9,44 | unknown |  |  |  | x |  |  |
| UniRef90_A5BA78 | Putative uncharacterized protein, Vitis vinifera | 59976 | 6,29 | unknown | x |  |  | x |  |  |
| UniRef90_A5BZ28 | Putative uncharacterized protein, Vitis vinifera | 11490 | 4,65 | unknown |  |  | x | x |  |  |
| UniRef90_A5C8M8 | Putative uncharacterized protein, Vitis vinifera | 50699 | 7,39 | unknown |  |  | x |  |  | x |
| UniRef90_A8I2H7 | Predicted protein, Chlamydomonas reinhardtii | 86620 | 5,62 | unknown |  |  |  |  | x |  |
| UniRef90_A9NMJ7 | Putative uncharacterized protein, Picea sitchensis | 35438 | 8,29 | unknown |  |  | x | x |  | x |
| UniRef90_A9RUR8 | Predicted protein, Physcomitrella patens | 110440 | 6,08 | unknown |  |  |  |  | x |  |
| UniRef90_A9RXN6 | Predicted protein, Physcomitrella patens | 29991 | 9,82 | unknown |  |  |  |  | x |  |
| UniRef90_A9S2G6 | Predicted protein, Physcomitrella patens | 109556 | 5,74 | unknown | x | x | x | x | x | x |
| UniRef90_A9TBV3 | SWIRM-domain protein, Physcomitrella patens | 214449 | 5,40 | unknown |  |  |  | x |  |  |
| UniRef90_A9TW25 | Predicted protein, Physcomitrella patens | 109179 | 5,74 | unknown | x |  |  | x | x | x |
| UniRef90_B4F9J6 | Putative uncharacterized protein, Zea mays | 16136 | 4,69 | unknown | x |  |  | x |  |  |
| UniRef90_B4FGN2 | Putative uncharacterized protein, Zea mays | 5497 | 4,43 | unknown | x |  |  |  |  |  |
| UniRef90_B4FL93 | Putative uncharacterized protein, Zea mays | 40487 | 4,82 | unknown | x |  |  |  |  |  |
| UniRef90_B4FRC6 | Putative uncharacterized protein, Zea mays | 38416 | 7,51 | unknown | x | x | x | x | x | x |
| UniRef90_B4FVA0 | Putative uncharacterized protein, Zea mays | 10219 | 9,92 | unknown |  |  |  |  |  |  |
| UniRef90_B4FVA0 | Putative uncharacterized protein, Zea mays | 10219 | 9,92 | unknown |  |  | x |  | x |  |
| UniRef90_B4FXI9 | Putative uncharacterized protein, Zea mays | 47689 | 4,74 | unknown | x |  | x |  | x | x |
| UniRef90_B4G1I1 | Putative uncharacterized protein, Zea mays | 43073 | 5,28 | unknown | x | x | x | x | x |  |
| UniRef90_B6SYG2 | Putative uncharacterized protein, Zea mays | 50021 | 5,27 | unknown | x |  | x |  | x | x |
| UniRef90_B6SZN3 | Putative uncharacterized protein, Zea mays | 27782 | 10,46 | unknown | x |  | x | x | x | x |
| UniRef90_B6UBR4 | Putative uncharacterized protein, Zea mays | 14504 | 9,84 | unknown | x | x | x | x | x | x |
| UniRef90_B6UDC4 | Putative uncharacterized protein, Zea mays | 12173 | 10,38 | unknown |  |  |  | x |  | x |
| UniRef90_B7EX82 | cDNA clone, Oryza sativa | 16767 | 4,57 | unknown | x |  | x | x |  |  |
| UniRef90_B7ZWX0 | Putative uncharacterized protein, Zea mays | 39130 | 6,21 | unknown |  |  | x |  |  | x |
| UniRef90_B7ZYG0 | Putative uncharacterized protein, Zea mays | 81314 | 5,91 | unknown | x | x | x |  |  | x |
| UniRef90_B8A1C7 | Putative uncharacterized protein, Zea mays | 14237 | 5,28 | unknown |  |  | x |  |  | x |
| UniRef90_B8A1T1 | Putative uncharacterized protein, Zea mays | 34906 | 5,49 | unknown | x |  |  |  | x |  |
| UniRef90_B8A260 | Putative uncharacterized protein, Zea mays | 3672 | 4,70 | unknown |  |  |  |  | x | x |
| UniRef90_B8AG04 | Putative uncharacterized protein, Oryza sativa | 21463 | 7,36 | unknown | x |  |  |  |  |  |
| UniRef90_B8B7Y5 | Putative uncharacterized protein, Oryza sativa | 71182 | 5,88 | unknown |  |  |  |  | x |  |
| UniRef90_B9DI26 | AT5G66190, Arabidopsis thaliana | 28751 | 5,06 | unknown |  |  | x |  | x |  |
| UniRef90_B9F4R1 | Putative uncharacterized protein, Oryza sativa | 106370 | 5,88 | unknown |  |  | x |  |  |  |
| UniRef90_B9FLH7 | Putative uncharacterized protein, Oryza sativa | 68451 | 5,30 | unknown |  |  |  | x |  |  |
| UniRef90_B9GL35 | Predicted protein, Populus trichocarpa | 85601 | 8,03 | unknown | x |  | x |  |  |  |
| UniRef90_B9GNZ6 | Predicted protein, Populus trichocarpa | 17146 | 5,70 | unknown |  |  |  |  | x |  |
| UniRef90_B9GTC2 | Predicted protein, Populus trichocarpa | 110041 | 6,27 | unknown | x |  | x | x | x | x |
| UniRef90_B9GU17 | Predicted protein, Populus trichocarpa | 77365 | 9,19 | unknown |  | x | x |  |  |  |
| UniRef90_B9H081 | Predicted protein, Populus trichocarpa | 41802 | 5,25 | unknown |  |  |  | x |  |  |
| UniRef90_B9IPS3 | Predicted protein, Populus trichocarpa | 66390 | 7,28 | unknown | x |  |  |  |  |  |
| UniRef90_B9MXF5 | Predicted protein, Populus trichocarpa | 40507 | 8,53 | unknown | x |  |  | x |  | x |
| UniRef90_B9P873 | Predicted protein, Populus trichocarpa | 12304 | 8,23 | unknown |  |  |  |  | x |  |
| UniRef90_B9SWE1 | Putative uncharacterized protein, Ricinus communis | 32600 | 7,63 | unknown |  | x | x |  |  | x |
| UniRef90_B9TKV9 | Putative uncharacterized protein, Ricinus communis | 17880 | 4,80 | unknown |  |  |  |  |  | x |
| UniRef90_C0HEX8 | Putative uncharacterized protein, Zea mays | 40293 | 5,42 | unknown |  |  | x |  |  |  |
| UniRef90_C0HHF9 | Putative uncharacterized protein, Zea mays | 68711 | 5,80 | unknown | x |  |  |  |  |  |
| UniRef90_C0HHF9 | Putative uncharacterized protein, Zea mays | 68711 | 5,80 | unknown |  | x | x | x | x |  |
| UniRef90_C0P4W9 | Putative uncharacterized protein, Zea mays | 38650 | 7,83 | unknown |  |  |  | x | x |  |
| UniRef90_C0PD28 | Putative uncharacterized protein, Zea mays | 53442 | 5,74 | unknown | x | x | x | x | x | x |
| UniRef90_C0PPC1 | Putative uncharacterized protein, Zea mays | 19803 | 5,39 | unknown |  | x | x |  |  | x |
| UniRef90_C1MYM2 | Predicted protein, Micromonas pusilla | 45927 | 4,64 | unknown | x |  |  |  |  |  |
| UniRef90_C4J0Z0 | Putative uncharacterized protein, Zea mays | 38813 | 12,55 | unknown |  | x | x |  | x | x |
| UniRef90_C4J389 | Putative uncharacterized protein, Zea mays | 72795 | 5,26 | unknown | x | x | x | x | x | x |
| UniRef90_C4J522 | Putative uncharacterized protein, Zea mays | 51574 | 7,84 | unknown | x | x | x | x | x | x |
| UniRef90_C4J6E4 | Putative uncharacterized protein, Zea mays | 26442 | 5,33 | unknown | x | x | x | x | x | x |
| UniRef90_C5WXC7 | Putative uncharacterized protein, Sorghum bicolor | 46929 | 7,91 | unknown |  | x | x |  | x | x |
| UniRef90_C5WXN3 | Putative uncharacterized protein, Sorghum bicolor | 40589 | 5,91 | unknown |  | x | x |  |  | x |
| UniRef90_C5WXN4 | Putative uncharacterized protein, Sorghum bicolor | 21907 | 9,57 | unknown |  |  | x |  |  | x |
| UniRef90_C5WYJ6 | Putative uncharacterized protein, Sorghum bicolor | 16597 | 8,02 | unknown | x |  | x |  |  |  |
| UniRef90_C5WYU1 | Putative uncharacterized protein, Sorghum bicolor | 5142 | 8,87 | unknown |  |  |  | x |  |  |
| UniRef90_C5X359 | Putative uncharacterized protein, Sorghum bicolor | 49706 | 6,33 | unknown | x | x | x | x | x | x |
| UniRef90_C5X498 | Putative uncharacterized protein, Sorghum bicolor | 85013 | 6,74 | unknown |  |  | x |  |  |  |
| UniRef90_C5X5K4 | Putative uncharacterized protein, Sorghum bicolor | 33350 | 6,55 | unknown | x | x |  |  | x | x |
| UniRef90_C5XD24 | Putative uncharacterized protein, Sorghum bicolor | 33369 | 8,69 | unknown |  | x |  |  | x |  |
| UniRef90_C5XG11 | Putative uncharacterized protein, Sorghum bicolor | 25191 | 9,35 | unknown | x |  | x | x | x | x |
| UniRef90_C5XI38 | Putative uncharacterized protein, Sorghum bicolor | 88088 | 5,22 | unknown | x | x |  |  |  |  |
| UniRef90_C5XY16 | Putative uncharacterized protein, Sorghum bicolor | 124775 | 5,61 | unknown |  |  | x |  | x |  |
| UniRef90_C5XYP5 | Putative uncharacterized protein, Sorghum bicolor | 84209 | 6,18 | unknown | x | x | x | x |  | x |
| UniRef90_C5Y210 | Putative uncharacterized protein, Sorghum bicolor | 41389 | 8,22 | unknown |  |  |  |  |  | x |
| UniRef90_C5Y397 | Putative uncharacterized protein, Sorghum bicolor | 114208 | 5,80 | unknown | x | x | x | x | x | x |
| UniRef90_C5Y853 | Putative uncharacterized protein, Sorghum bicolor | 15416 | 9,68 | unknown |  |  |  | x |  |  |
| UniRef90_C5Y8Y2 | Putative uncharacterized protein, Sorghum bicolor | 80854 | 5,97 | unknown | x | x | x | x | x | x |
| UniRef90_C5YCL4 | Putative uncharacterized protein, Sorghum bicolor | 82952 | 6,04 | unknown | x | x | x | x |  | x |
| UniRef90_C5YGD9 | Putative uncharacterized protein, Sorghum bicolor | 28730 | 5,74 | unknown | x |  |  | x |  |  |
| UniRef90_C5YQN5 | Putative uncharacterized protein, Sorghum bicolor | 72459 | 5,15 | unknown | x | x | x | x | x | x |
| UniRef90_C5Z4E5 | Putative uncharacterized protein, Sorghum bicolor | 39434 | 7,59 | unknown |  | x | x | x | x | x |
| UniRef90_C5Z8K3 | Putative uncharacterized protein, Sorghum bicolor | 20811 | 9,58 | unknown |  |  |  | x |  |  |
| UniRef90_C6T7B0 | Putative uncharacterized protein, Glycine max | 48510 | 5,42 | unknown | x |  |  |  |  |  |
| UniRef90_C6T8W8 | Putative uncharacterized protein, Glycine max | 28429 | 7,96 | unknown | x |  |  |  |  |  |
| UniRef90_C6TFG7 | Putative uncharacterized protein, Glycine max | 34875 | 8,15 | unknown |  | x | x |  | x | x |
| UniRef90_C6TKH0 | Putative uncharacterized protein, Glycine max | 31640 | 6,41 | unknown | x |  |  |  |  |  |
| UniRef90_Q08056 | Putative carbonic anhydrase homolog, Zea mays | 7312 | 7,31 | unknown |  |  |  | x | x |  |
| UniRef90_Q0H633 | Putative chloroplast chlorophyll A B binding protein type II, Sorghum bicolor | 11894 | 4,58 | unknown |  |  |  | x |  |  |
| UniRef90_Q10JY1 | Retrotransposon protein, Oryza sativa | 76960 | 9,08 | unknown | x | x |  | x |  |  |
| UniRef90_Q2QVG7 | Putative uncharacterized protein, Oryza sativa | 30428 | 9,09 | unknown |  |  | x |  |  |  |
| UniRef90_Q2QXU4 | Putative uncharacterized protein, Oryza sativa | 18592 | 9,84 | unknown |  |  |  | x |  |  |
| UniRef90_Q339E4 | Putative uncharacterized protein, Oryza sativa | 22361 | 9,94 | unknown |  |  |  |  | x |  |
| UniRef90_Q5N7F1 | Putative uncharacterized protein, Oryza sativa | 14728 | 8,00 | unknown |  | x | x |  | x | x |
| UniRef90_Q5QNA5 | Os01g0208700 protein, Oryza sativa | 114125 | 5,70 | unknown |  |  |  |  | x |  |
| UniRef90_Q5VS63 | Putative polygalacturonase, Oryza sativa | 51393 | 5,93 | unknown | x |  |  |  |  |  |
| UniRef90_Q5Z6G3 | Putative RNA polymerase I II and III 16.5 kDa subunit, Oryza sativa | 43542 | 9,26 | unknown |  |  | x | x |  | x |
| UniRef90_Q6ERF5 | Putative uncharacterized protein, Oryza sativa | 6621 | 8,55 | unknown |  |  |  |  | x |  |
| UniRef90_Q6Z8I7 | Os02g0752200 protein, Oryza sativa | 84253 | 6,33 | unknown |  |  | x |  |  |  |
| UniRef90_Q6ZFJ4 | Os02g0103800 protein, Oryza sativa | 38723 | 7,82 | unknown | x | x | x | x | x | x |
| UniRef90_Q8LM49 | Putative uncharacterized protein, Oryza sativa | 8895 | 8,31 | unknown |  |  | x |  |  |  |
| UniRef90_Q8RW05 | Putative phosphoenolpyruvate carboxylase, Sorghum bicolor | 45591 | 5,37 | unknown | x |  | x | x | x | x |
| UniRef90_Q8RW22 | Putative phosphoenolpyruvate carboxylase, Paspalidium geminatum | 45756 | 5,55 | unknown |  |  |  |  | x |  |
| UniRef90_Q8S2R2 | Putative phosphoenolpyruvate carboxylase, Aristida mauritiana | 43753 | 5,26 | unknown | x | x | x |  | x |  |
| UniRef90_Q8S2Z8 | Putative C4 phosphoenolpyruvate carboxylase, Setaria italica | 110000 | 5,80 | unknown | x | x | x | x | x | x |
| UniRef90_Q9LSC0 | Genomic DNA, Arabidopsis thaliana | 38419 | 9,57 | unknown | x |  |  |  |  |  |
| UniRef90_Q9SNK3 | EST C74302 E30840, Oryza sativa | 47081 | 6,22 | unknown | x |  |  |  |  |  |
| UniRef90_Q9SUL1 | AT4g16190, Arabidopsis thaliana | 41237 | 6,52 | unknown |  |  |  |  | x | x |
| UniRef90_Q9XET0 | Putative uncharacterized protein, Glycine max | 15088 | 9,42 | unknown | x |  |  |  |  |  |
| UniRef90_Q9ZW79 | Putative clathrin binding protein Epsin, Arabidopsis thaliana | 30781 | 9,55 | unknown |  |  | x | x |  |  |
