# Supplementary material for: Comparative evaluation of extraction methods for apoplastic proteins from maize leaves
Source: Plant Methods. 2011 Dec 22;7:48. doi: 10.1186/1746-4811-7-48 (PMC3284877; doi:10.1186/1746-4811-7-48)
Supplement: Additional file 4 — Proteins identified in apoplast extracts of all six infiltration solutions. [file 1746-4811-7-48-S4.DOC]

| **Entry** | **Description** |
| --- | --- |
| UniRef90_A2XJC7 | Putative uncharacterized protein, Oryza sativa |
| UniRef90_A7DXA5 | Phosphoenolpyruvate carboxylase, Panicum capillare |
| UniRef90_A7DXE1 | Phosphoenolpyruvate carboxylase, Tristachya leucothrix |
| UniRef90_A8ASG2 | Phosphoenolpyruvate carboxylase, Aloe arborescens |
| UniRef90_A9S2G6 | Predicted protein, Physcomitrella patens |
| UniRef90_B4FRC6 | Putative uncharacterized protein, Zea mays |
| UniRef90_B6T391 | Lichenase 2, Zea mays |
| UniRef90_B6TEW2 | Ferredoxin NADP reductase, Zea mays |
| UniRef90_B6TG95 | Vignain, Zea mays |
| UniRef90_B6UBR4 | Putative uncharacterized protein, Zea mays |
| UniRef90_C0PD28 | Putative uncharacterized protein, Zea mays |
| UniRef90_C4J389 | Putative uncharacterized protein, Zea mays |
| UniRef90_C4J522 | Putative uncharacterized protein, Zea mays |
| UniRef90_C4J6E4 | Putative uncharacterized protein, Zea mays |
| UniRef90_C5X359 | Putative uncharacterized protein, Sorghum bicolor |
| UniRef90_C5Y397 | Putative uncharacterized protein, Sorghum bicolor |
| UniRef90_C5Y8Y2 | Putative uncharacterized protein, Sorghum bicolor |
| UniRef90_C5YQN5 | Putative uncharacterized protein, Sorghum bicolor |
| UniRef90_D2T2H9 | Phosphoenolpyruvate carboxylase, Holcus lanatus |
| UniRef90_D3KY86 | Phosphoenolpyruvate carboxylase, Ananas comosus |
| UniRef90_P11155 | Pyruvate phosphate dikinase 1, Zea maize |
| UniRef90_P93518 | PRm 3, Zea maize |
| UniRef90_Q198V8 | Phosphoenolpyruvate carboxylase, Suaeda eltonica |
| UniRef90_Q52NW0 | Phosphoenolpyruvate carboxylase, Echinochloa crus |
| UniRef90_Q6TM44 | Germin-like protein, Zea mays |
| UniRef90_Q6ZFJ4 | Os02g0103800 protein, Oryza sativa |
| UniRef90_Q8S2Z8 | Putative C4 phosphoenolpyruvate carboxylase, Setaria italica |
| UniRef90_Q9XE93 | Exhydrolase II, Zea mays |
